# Improvement of predictive accuracies of functional outcomes after subacute stroke inpatient rehabilitation by machine learning models

**DOI:** 10.1371/journal.pone.0286269

**Published:** 2023-05-26

**Authors:** Yuta Miyazaki, Michiyuki Kawakami, Kunitsugu Kondo, Masahiro Tsujikawa, Kaoru Honaga, Kanjiro Suzuki, Tetsuya Tsuji

**Affiliations:** 1 Department of Physical Rehabilitation, National Center Hospital, National Center of Neurology and Psychiatry, Tokyo, Japan; 2 Department of Rehabilitation Medicine, Tokyo Bay Rehabilitation Hospital, Chiba, Japan; 3 Department of Rehabilitation Medicine, Keio University School of Medicine, Tokyo, Japan; 4 Department of Rehabilitation Medicine, Juntendo University Graduate School of Medicine, Tokyo, Japan; 5 Department of Rehabilitation Medicine, Waseda Clinic, Miyazaki, Japan; Jahangirnagar University, BANGLADESH

## Abstract

**Objectives:**

Stepwise linear regression (SLR) is the most common approach to predicting activities of daily living at discharge with the Functional Independence Measure (FIM) in stroke patients, but noisy nonlinear clinical data decrease the predictive accuracies of SLR. Machine learning is gaining attention in the medical field for such nonlinear data. Previous studies reported that machine learning models, regression tree (RT), ensemble learning (EL), artificial neural networks (ANNs), support vector regression (SVR), and Gaussian process regression (GPR), are robust to such data and increase predictive accuracies. This study aimed to compare the predictive accuracies of SLR and these machine learning models for FIM scores in stroke patients.

**Methods:**

Subacute stroke patients (N = 1,046) who underwent inpatient rehabilitation participated in this study. Only patients’ background characteristics and FIM scores at admission were used to build each predictive model of SLR, RT, EL, ANN, SVR, and GPR with 10-fold cross-validation. The coefficient of determination (R^2^) and root mean square error (RMSE) values were compared between the actual and predicted discharge FIM scores and FIM gain.

**Results:**

Machine learning models (R^2^ of RT = 0.75, EL = 0.78, ANN = 0.81, SVR = 0.80, GPR = 0.81) outperformed SLR (0.70) to predict discharge FIM motor scores. The predictive accuracies of machine learning methods for FIM total gain (R^2^ of RT = 0.48, EL = 0.51, ANN = 0.50, SVR = 0.51, GPR = 0.54) were also better than of SLR (0.22).

**Conclusions:**

This study suggested that the machine learning models outperformed SLR for predicting FIM prognosis. The machine learning models used only patients’ background characteristics and FIM scores at admission and more accurately predicted FIM gain than previous studies. ANN, SVR, and GPR outperformed RT and EL. GPR could have the best predictive accuracy for FIM prognosis.

## Introduction

Stroke is one of the leading causes of acquired disability [1]. The incidence and mortality of stroke in high-income countries have decreased, whereas disability-adjusted life-years lost have increased significantly [2]. Functional recovery of motor disability and cognitive dysfunction are associated with discharge destination [3, 4]. Therefore, early predictions of functional recovery will give post-stroke patients in the subacute stage relevant information to plan discharge destinations [5], and it becomes a good clinical decision-making tool for patients and families.

A previous study reported that clinical findings on admission could predict post-stroke functional recovery with the Functional Independence Measure (FIM) [6, 7]. The FIM was designed to evaluate motor disability and cognitive dysfunction in activities of daily living (ADL) [8]. FIM scores on admission could predict discharge FIM scores by multiple linear regression [9]. Additional clinical indicators on admission, such as the Trunk Impairment Scale [10], Stroke Impairment Assessment Set [11], and comorbidity index [12], improved the predictive accuracy of discharge FIM scores. These studies built stepwise linear regression (SLR) models, but noisy and non-linear datasets in stroke neurorehabilitation could decrease predictive accuracies [13].

Machine learning could potentially build more accurate prognostic models than SLR, because it is robust to complex non-linear data [14, 15]. Several studies have suggested that machine learning could predict functional outcomes in acute/subacute stroke patients, with the Barthel index [16] and the modified Rankin Scale score [17, 18]. The discharge FIM scores were also predicted with an artificial neural network (ANN) [19] and support vector regression (SVR) [20]. ANN is one of the famous machine learning algorithms used for artificial intelligence and is widely used in regression and classification in clinical fields [21]. ANN consists of artificial neurons (nodes) and layers consisting of a group of artificial neurons. ANN is designed to optimize weighted neural connections to predict outcomes [13]. SVR has also been widely used for classification and regression because it can build predictive models with non-linear variables by kernel functions [22, 23]. ANN and SVR have better predictive accuracies than conventional linear statistical analysis [24]. Classification and regression trees(CART) are widely used to construct prediction models from data, and regression trees(RT), which is a part of CART, can analyze both of linear and non-linear data to build regression models [25]. CART is widely used to analyze in the medical fields [26, 27]. The advantage of CART is to determine thresholds and more easily understand the prognostic models than other machine learning algorithms [28]. Ensemble learning(EL) can improve predictive accuracy by combining the weak classification models, and boosting and bagging are well-known algorithms to construct the predictive models [29]. The previous study also reported that the Regression Tree Ensemble learning can be used to analyze the post-stroke functional recovery of upper limbs [30]. A review article reported that an increasing number of studies have reported the prediction of functional outcomes of stroke in recent years [31]. According to another review article, there have been six studies of the modified Rankin Scale and one study of the FIM [32]. Therefore, machine learning algorithms have not been adequately considered in FIM prognosis research.

Gaussian process regression (GPR) can predict an output variable based on the similarities between input variables, and it is robust to noisy data [33]. SLR assumes linear or exponential models, but clinical data do not necessarily satisfy the assumption [34]. SLR and other regression algorithms build predictive models to decrease the difference between original and predicted data and predict the best value; in contrast, GPR can also predict the probabilistic functional outcome with the predicted distribution. The predicted distribution could provide a comprehensive summary that is suitable for predicting prognosis in clinical fields [34]. Therefore, GPR has been used in clinical fields in recent studies [35]. For example, GPR can accurately predict the Functional Ability Scales in head trauma patients with wearable sensors [36] and functional outcomes after stroke with magnetic resonance images [37, 38].

Although previous studies have assumed a linear model for the FIM score, it is essential to consider that the FIM score is strictly nonlinear, and that clinical data are subject to noise. For example, even if the FIM score at admission is the same, it is necessary to consider a certain range in FIM scores at discharge. Therefore, we think that assuming a linear model will predict a poor fit when creating a prediction model using FIM scores. This study used SLR as a conventional regression method, and RT, EL, ANN, and SVR were used as previously reported machine learning methods. GPR was also used as a novel prognostic model for discharge FIM scores. The present study aimed to compare the predictive accuracies of SLR and machine learning methods (RT, EL, ANN, SVR, and GPR) for discharge FIM scores in stroke patients.

## Methods

### Study design

This observational, retrospective study was approved by the Tokyo Bay Rehabilitation Hospital’s Institutional Review Board (267–2). This study was conducted in accordance with the principles of the Declaration of Helsinki [39].

### Participants

**Fig 1:**
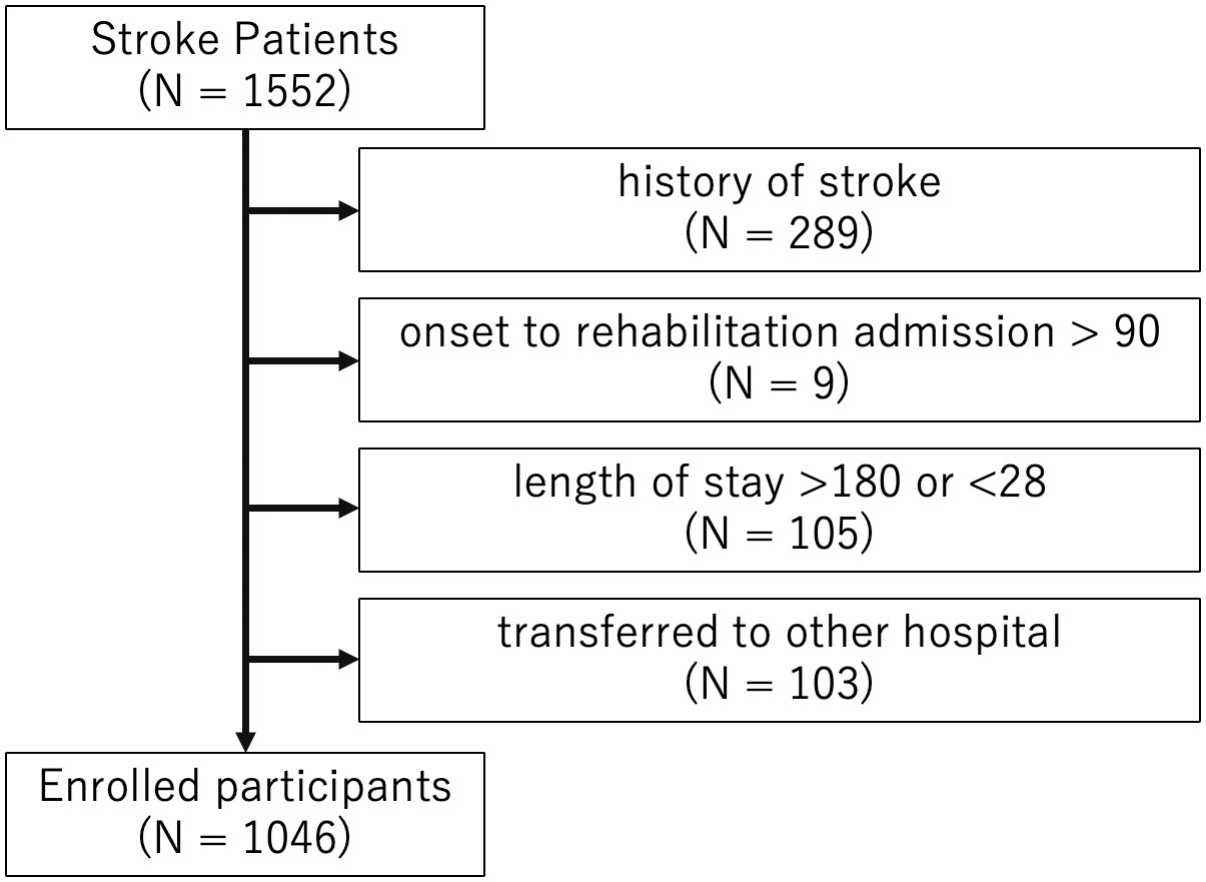
Exclusion criteria.

A total of 1,552 subacute stroke patients were admitted to Tokyo Bay Rehabilitation Hospital between March 1^st^, 2015, and September 30^th^, 2019. After acute treatments, most subacute stroke patients usually transfer to rehabilitation hospitals to receive intensive rehabilitation in Japan, and Tokyo Bay Rehabilitation Hospital is one of them. The inclusion criteria were (1) the first unilateral ischemic or hemorrhagic stroke, (2) length between admission day and onset was less than 90 days (days since onset), (3) length of stay between 28 and 180 days, and (4) no history of transfer to an acute hospital. A total of 1,046 eligible patients were enrolled in the present study (Fig 1). Informed consent was obtained in the form of opt-out on the Tokyo Bay Rehabilitation hospital’s website to exclude people who refused participation. All participants received conventional physical, occupational, and speech therapy for 3 hours daily. Trained nurses recorded participants’ FIM scores every 2 weeks, and these data were stored in an electronic medical database.

### Data acquisition

Participants’ data were obtained from an electronic medical record database at Tokyo Bay Rehabilitation hospital that included age, sex, days since onset, admission and discharge FIM scores [8], history of stroke, and transfer to other hospitals (Table 1).

**Table 1:** Participants’ data.

	Mean ± SD (Min-Max), n(%)
Age (y)	69.0 ± 14.4 (13–98)
Sex	
Female	459 (43.9%)
Male	587 (56.1%)
Days since stroke onset	33.9 ± 12.5 (10–86)
Stroke type	
Ischemic	584 (55.8%)
Cerebral hemorrhage	382 (36.5%)
Subarachnoid hemorrhage	80 (7.7%)
Admission FIM motor score	45.1 ± 21.8 (13–91)
Discharge FIM motor score	70.7 ± 22.7 (13–91)
FIM motor score gain	25.9 ± 15.2 (-17–72)
Admission FIM cognitive score	22.7 ± 8.8 (5–35)
Discharge FIM cognitive score	27.2 ± 7.9 (5–35)
Admission FIM total score	67.8 ± 28.7 (18–126)
Discharge FIM total score	97.9 ± 29.4 (18–126)

N = 1,046, SD: standard deviation, FIM: Functional Independence Measure

The Japanese version of FIM (version 3.0) [7, 40], which has culturally relevant modifications for some of the items, was used [41, 42]. In this study, we focused on comparing the accuracies of each machine learning model. If we adopted more clinical indicators than previous research and compared the accuracies of each machine learning model, we cannot assess whether machine learning or additional clinical indicators contribute more to accuracies, so we adopted only a minimum of these basic clinical indicators.

### Model development and statistical analysis

In the present study, raw FIM scores at discharge were some of the rehabilitation outcomes, and FIM motor scores, cognitive scores, and total scores at discharge were evaluated. FIM gain, defined as the change in the score between admission and discharge [43], was also examined in the present study. FIM motor gain, cognitive gain, and total gain were calculated. A previous study [44] evaluated the coefficient of determination (R^2^) between actual FIM scores and FIM scores predicted by predictive models. A previous study also evaluated Root Mean Squared Error (RMSE) between actual and predicted FIM scores [20]. Therefore, R^2^ values and RMSE of FIM motor scores, FIM cognitive scores, FIM total scores, FIM motor gain, FIM cognitive gain, and FIM total gain were compared among predictive models in the present study.

A forward-backward Stepwise linear regression (SLR) was used as a conventional statistical method to predict functional outcomes in this study [44]. P-value of < 0.05 was used for the declaration of statistical significance. In addition, five machine learning algorithms, RT [25], EL [30], SVR [22], ANN [13], and GPR [45], were used. Previous studies reported the prediction of functional outcomes after stroke with ANN [19] and SVR [20]. To our best knowledge, this is the first time that GPR has been used as a novel method for predicting FIM scores.

The predictor variables were age, days since onset, and admission FIM scores (motor, cognitive, and total scores). Each prediction model was fitted to discharge FIM motor scores, discharge FIM cognitive scores, discharge FIM total scores, FIM motor gain, FIM cognitive gain, and FIM total gain. Statistical analyses were performed, and predictive models were built with MATLAB software, version 2022a (MathWorks, Natick, MA, USA).

Overlearning is widely known in machine learning, especially in ANN [46]. If the machine learning models have no restrictions to learn features, they can “memorize” all samples and improve the accuracy of training data sets or similar data sets. However, the predictive accuracy of dissimilar data sets decreases when overlearning occurs. Therefore, the prevention of overlearning is important to improve generalization performance [46]. The data were first divided into a training data set (80%) and a test data set (20%) [47] to evaluate generalization performance before learning.

**Fig 2:**
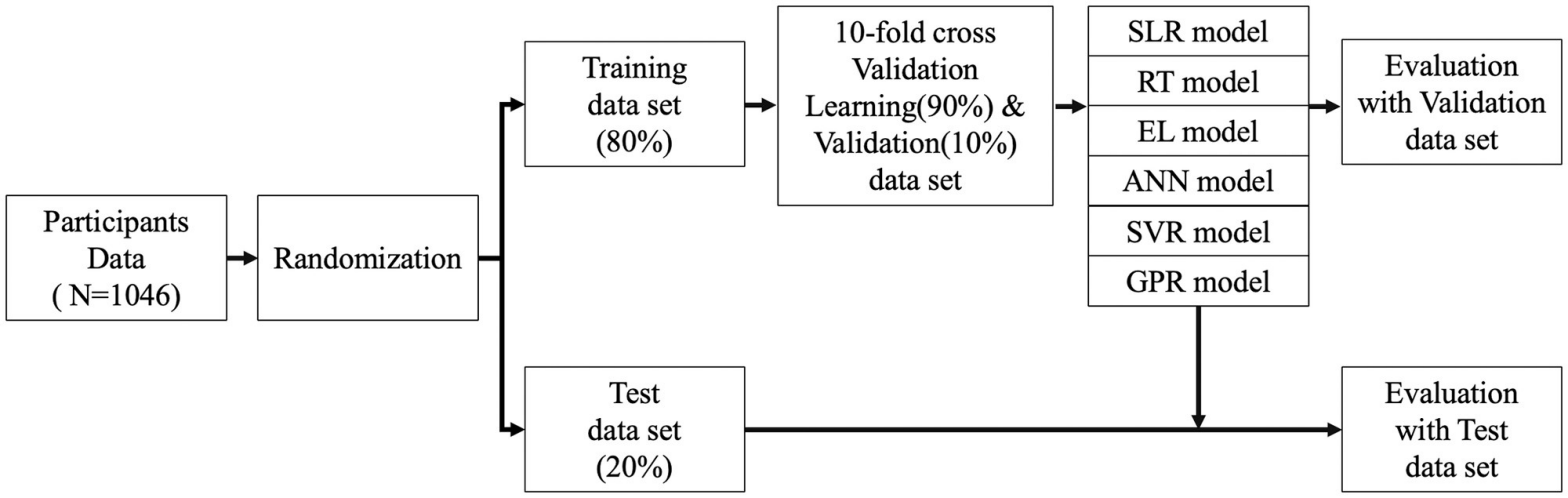
The flow chart of model development and the validation process. SLR: Stepwise Linear Regression, RT: Regression Tree, EL: Ensemble Learning, ANN: Artificial Neural Network, SVR: Support Vector Regression, GPR: Gaussian Process Regression.

The training data set was used to develop predictive models with 10-fold cross-validation. In the 10-fold cross validation [48], the training data set was randomly split into 10 groups, 9 groups were used as learning data sets, and the remaining group was used as a validation data set. This process was repeated 10 times (Fig 2). RMSE was used as a performance indicator in the present study. Hyperparameters were automatically assigned by MATLAB software through 10-fold cross-validation. After building the predictive models, each model was evaluated with test data sets. Predictive accuracies of each model were compared with adjusted R^2^ and RMSE between actual and predicted values.

## Results

In this study, machine learning models (RT, EL, ANN, SVR, and GPR) improved the predictive accuracies of FIM prognosis compared to SLR. The predictive performances of each model in validation and test data sets of FIM scores are presented in Table 2, and those of FIM gain are presented in Table 3. The coefficients of the SLR models are presented in S1 Table.

**Table 2:** Results for validation and test data sets of FIM scores.

	FIM motor scores			
	Validation		Test	
	R^2^	RMSE	R^2^	RMSE
SLR	0.67	13.057	0.70	12.625
RT	0.78	11.341	0.75	11.497
EL	0.77	10.899	0.78	10.778
ANN	0.77	10.808	0.81	10.079
SVR	0.78	10.517	0.80	10.262
GPR	0.79	10.251	0.81	10.023
	FIM cognitive scores			
	Validation		Test	
	R^2^	RMSE	R^2^	RMSE
SLR	0.71	4.199	0.74	4.067
RT	0.72	4.185	0.73	4.157
EL	0.73	4.059	0.72	4.225
ANN	0.70	4.274	0.72	4.193
SVR	0.71	4.268	0.74	4.088
GPR	0.76	3.880	0.74	4.053
	FIM total scores			
	Validation		Test	
	R^2^	RMSE	R^2^	RMSE
SLR	0.72	15.475	0.69	16.536
RT	0.77	14.219	0.77	14.236
EL	0.78	13.667	0.75	14.685
ANN	0.80	13.043	0.80	13.389
SVR	0.81	12.799	0.78	13.811
GPR	0.82	12.552	0.80	13.286

FIM: Functional Independence Measure, SLR: Stepwise Linear Regression, RT: Regression Tree, EL: Ensemble Learning, ANN: Artificial Neural Network, SVR: Support Vector Regression, GPR: Gaussian Process Regression, R^2^: coefficient of determination, RMSE: Root Mean Squared Error

**Table 3:** Results for validation and test data sets of FIM gain.

	FIM motor gain			
	Validation		Test	
	R^2^	RMSE	R^2^	RMSE
SLR	0.26	13.136	0.24	12.894
RT	0.49	10.954	0.41	11.320
EL	0.52	10.551	0.43	11.164
ANN	0.52	10.602	0.49	10.583
SVR	0.53	10.444	0.50	10.465
GPR	0.55	10.310	0.49	10.598
	FIM cognitive gain			
	Validation		Test	
	R^2^	RMSE	R^2^	RMSE
SLR	0.29	4.067	0.25	4.584
RT	0.28	4.080	0.36	4.211
EL	0.33	3.944	0.39	4.127
ANN	0.37	3.830	0.38	4.156
SVR	0.35	3.882	0.36	4.219
GPR	0.36	3.854	0.37	4.181
	FIM total gain			
	Validation		Test	
	R^2^	RMSE	R^2^	RMSE
SLR	0.23	15.752	0.22	15.735
RT	0.38	14.171	0.48	12.776
EL	0.45	13.356	0.51	12.431
ANN	0.47	13.033	0.50	12.549
SVR	0.47	13.056	0.51	12.485
GPR	0.48	12.935	0.54	12.106

FIM: Functional Independence Measure, SLR: Stepwise Linear Regression, RT: Regression Tree, EL: Ensemble Learning, ANN: Artificial Neural Network, SVR: Support Vector Regression, GPR: Gaussian Process Regression, R^2^: coefficient of determination, RMSE: Root Mean Squared Error

### Prediction of FIM scores

Machine learning methods outperformed SLR to predict FIM motor and FIM total scores. Machine learning improved predictive accuracies (R^2^ = 0.77–0.79, RMSE = 10.251–11.341) of FIM motor scores in validation data sets compared to SLR (R^2^ = 0.67 and RMSE = 13.057). Predictive accuracies of test data sets for FIM motor scores were better than those of validation data sets except RT. GPR has the best predictive accuracies for FIM motor scores. In contrast, the predictive accuracies of FIM cognitive scores showed no differences between SLR and machine learning models. GPR has the best R^2^ and RMSE as well as FIM motor scores. In FIM total scores, R^2^ and RMSE of machine learning improved more than SLR. Among the machine learning, ANN, SVR, GPR tended to perform better than RT and EL. GPR had also the best predictive accuracies in all models. No overlearning was observed in our study because big differences in R^2^ and RMSE between the validation and test data sets were not observed.

### Prediction of FIM gain

Machine learning also improved the predictive accuracy of FIM gain more than SLR. Machine learning (R^2^ = 0.41–0.50, RMSE 10.465–11.320) showed improvements over SLR (R^2^ = 0.24, RMSE = 12.849) for FIM motor gain. Comparing the prognostic accuracy between the validation and test data sets, RT and EL showed a significant decrease, whereas ANN, SVR, and GPR decreased only slightly. The present results showed that ANN, SVR, and GPR outperformed SLR, RT, and EL to predict FIM motor gain. To predict FIM cognitive gain, machine learning also outperformed SLR. RT and EL had bigger differences of accuracies between validation and test data sets compared those of ANN, SVR, and GPR. Therefore, ANN, SVR, and GPR showed stable prognostic accuracy between validation and test data sets for FIM cognitive gain. For FIM total gain, machine learning showed better R^2^ and RMSE than SLR. RT and EL showed larger difference in predictive accuracy between validation and test data sets than those of ANN, SVR, and GPR. GPR had the best prognostic accuracy (R^2^ = 0.54, RMSE = 12.106) among the predictive models of FIM total gain.

## Discussion

The present study aimed to compare the predictive accuracies of SLR and machine learning methods (RT, EL, ANN, SVR, and GPR) for discharge FIM scores in subacute stroke patients. Machine learning models outperformed SLR models to predict FIM scores and FIM gain, excluding FIM cognitive scores. The result notably suggested that machine learning models increased the predictive accuracies of FIM gain compared to SLR models.

### Comparison of FIM scores between the present study and previous studies

Machine learning models potentially improve the prognostic accuracies of FIM scores at discharge compared to the linear regression model because machine learning models can adapt to complicated non-linear data. The type of model and level of regularization would affect R^2^; therefore, the use of R^2^ for model comparison with different data sets needs careful attention [49]. A previous review reported that the mean R^2^ for discharge FIM motor scores was 0.65 (range 0.35 to 0.82) on multiple linear regression analysis [44]. Therefore, the present machine learning models (R^2^ = 0.75–0.81) only using patients’ backgrounds and FIM scores at admission had better predictive accuracies than most of the previous research. Moreover, the present study showed that the R^2^ of SLR for the discharge FIM motor score was 0.67 for the validation data set and 0.70 for the test data set. This result implied that the present participants were not easier to predict FIM prognosis and were not more suitable for SLR than those in previous studies. A previous study reported that SVR had good prognostic accuracy for discharge FIM motor scores (RMSE = 26.79) with 55 participants [20]. The SVR model in the present study showed better performance (RMSE = 10.262) than in the previous study. One possible explanation for this difference was the sample size. Machine learning methods require large sample sizes to achieve the best prediction accuracy [50], and recommended sample sizes are several hundred [51]. Previous studies’ sample sizes might not have been sufficient to achieve maximum accuracies; then, the accuracies could decrease because of overlearning, which is one of the problems that decrease machine learning accuracies. In contrast, the present sample size was a total of 1,046 participants, with 753 participants for learning data sets, which should be large enough, and maximum accuracy was achieved. Overlearning was negligible to predict FIM scores because the R^2^ values of the test data sets did not show a large decrease from those of the validation data sets. Therefore, the present models could have better accuracies with generalization performance.

### Comparison of FIM gain between the present study and previous studies

The present study also suggested that machine learning models more accurately predicted the FIM gain than SLR. A review article reported that predictive accuracies for FIM motor gain (R^2^ for FIM motor gain was 0.22, range 0.08 to 0.40) were lower than those for discharge FIM motor scores [44]. R^2^ for FIM motor gain of the five machine learning models ranged from 0.41 to 0.55, and it was higher than the 0.24 of SLR in the present study. To our best knowledge, our machine learning models are better than previously published models in predicting FIM motor gain.

### Comparison of predictive accuracies among machine learning models

GPR showed better predictive accuracies for FIM motor and total scores, and FIM total gain than the other five models. The RMSE of GPR for FIM total scores was 13.286, the best accuracy of all models. The RMSE of GPR for FIM total gain (RMSE = 12.106) was also the best of all models. Therefore, GPR is suitable for predicting FIM total scores and FIM total gain. A previous study suggested that GPR had better predictive accuracy after spinal cord injury than SVR and SLR [34]. The present result was compatible with the previous study and is the first report of using GPR to predict FIM. In addition, GPR had the best RMSE and might have the best prediction accuracy of the three machine learning methods.

In contrast, machine learning methods did not improve the predictive accuracies for FIM cognitive scores. One possible reason was that the same FIM cognitive scores had more diversity than the same motor scores, because FIM cognitive scores could not include patients’ background characteristics. Since machine learning methods built the prognostic models with the same numbers as the same cases, it is thought that the accuracy of prognosis prediction decreases when there are different cases with the same numbers. Therefore, most previous research reported only the FIM motor scores or FIM total scores, excluding cognitive scores.

One of the possible reasons for the improved accuracies of machine learning (RT, EL, SVR, ANN, and GPR) over SLR is that machine learning can handle non-linear data. Neurorehabilitation data could be prone to consist of complex non-linear data, and they are also prone to noise contamination due to human error and lack of data [13]. ANN is designed to consider non-predefined and nonlinear relationships that conventional analyses cannot recognize [52, 53]. SVR [22] and GPR [33] can be treated as linear models using kernel functions. Nonlinear analysis may be one of the reasons for improved prognostic accuracy. ANN [13] and SVR [54] are also robust to noise. In particular, the GPR model is characterized by its resistance to noise. It was used in electrocardiograms with much noise and improved clinical diagnosis accuracy [35], and it was also used for big data in epidemiology [55]. Among machine learning systems, SVR, ANN, and GPR are designed to be robust to noise. Therefore, they outperformed RT and EL.

The present machine learning models using only age, days since onset, and FIM scores outperformed multiple linear regression models that previous studies reported with other clinical indicators. Time and human resources at admission are usually limited; therefore, a simplified method is required to predict prognosis. In the present study, only FIM scores at admission without other clinical indicators were deliberately used to save time and to be easily used in clinical practice. Previous studies reported that the addition of functional impairment, such as the Trunk Impairment Scale [10], Stroke Impairment Assessment Set [11], comorbidity index [12], and nutritional conditions [56], to the FIM scores improved prediction accuracy. NIH Stroke scale is also well known as a good predictor in acute phase [57], but we did not consider it because we think it is not suitable for subacute stroke patients who enrolled in our study. It has been reported that the integration of conventional clinical indicators and neuroimaging biomarkers has significantly improved predictive accuracy [58], and the addition of neuroimaging to this study will be expected to further improve predictive accuracy. Further studies with specific deep learning tools for neuroimaging biomarkers have the potential to improve prediction accuracies for subacute stroke patients. Predictive accuracy is expected to be improved by incorporating clinical indicators in future studies.

### Limitations of this study

The first limitation of the present study is that it was an observational, retrospective study at a single center; therefore, one should consider over-adaptation to a single center and adaptation to multiple centers in a future study. Second, the present study did not include other clinical indicators such as SIAS and TIS to save time and be easily used in practice, and these indicators could increase prognostic accuracy. Moreover, the present study did not include neuroimaging biomarkers such as acute stroke volume, arterial occlusion grade, ischemic penumbra size, etc. Further larger, multicenter studies should be conducted that include clinical indicators and imaging biomarkers to confirm these preliminary results. Third, the present study did not contain enough cases to examine deep learning, and deep learning was not considered. If the number of features and cases increases, deep learning will be considered, which is expected to have higher prediction accuracy than the machine learning models used in the present study. Fourth, machine learning models except RT could not show the contribution of each explanatory variable to improving predictive accuracies because machine learning is a black box, unlike SLR.

## Conclusions

The results of the present study suggest that machine learning could improve the predictive accuracy of discharge FIM scores and FIM gain compared to SLR with the same stroke patients’ data set. Machine learning models with only admission FIM scores had better predictive accuracy than previous studies with other clinical indicators; therefore, they had the potential to be easily used in daily medical practice to improve prognostic accuracy with other clinical indicators. On comparison of machine learning algorithms, ANN, SVR, and GPR outperformed RT and EL. This study is the first to have used GPR to predict FIM, and GPR had better predictive accuracies for FIM total scores and FIM total gain than other models. In addition, this is the first study with enough participants to build machine learning models for predicting FIM, and overlearning did not occur.

## Supporting information

S1 Table Coefficients of SLR models. SE: standard error; Since Onset: days since onset, FIM: Functional Independence Measure. The aim of our study was to compare the predictive accuracies of a conventional stepwise linear regression (SLR) model and five machine learning models, Regression Tree, Ensemble Learning, Artificial Neural Network, Support Vector Regression, and Gaussian Process Regression. This study built the prognostic models for Activities of Daily Living (ADL) with the Functional Independence Measure (FIM), one of the methods for evaluating ADL. Discharge FIM motor scores, FIM cognitive scores, and FIM total scores were predicted. FIM gain is calculated by subtracting the scores at admission from those at the time of discharge. FIM motor gain, FIM cognitive gain, and FIM total gain were also predicted. A total of 1,046 subacute stroke patients who underwent inpatient rehabilitation participated in the present study. Patient information including age, sex, days since onset, admission and discharge FIM scores, a history of stroke, and transfer to other hospitals was gathered. Statistical analysis was performed with MATLAB software, version 2022a (The Mathworks, Natick, MA, USA). These predictive models were built with these participants’ information and 10-fold cross-validation. S1 Table shows the factors selected by the SLR model, and each value shows the intercept and coefficients.
(DOCX)

## Abbreviations

ANN: artificial neural network
EL: Ensemble Learning
FIM: functional independence measure
GPR: Gaussian process regression
R: Pearson’s correlation coefficient
R^2^: coefficient of determination
RMSE: root mean square errors
RT: Regression Tree
SLR: Stepwise linear regression
SVR: support vector regression

## References

[1] Furie K. Epidemiology and Primary Prevention of Stroke. Continuum (Minneapolis, Minn). 2020; 26(2): 260–267. doi: 10.1212/CON.0000000000000831 32224751

[2] Krishnamurthi RV, Feigin VL, Forouzanfar MH, Mensah GA, Connor M, Bennett DA, et al. Global and regional burden of first-ever ischaemic and haemorrhagic stroke during 1990–2010: findings from the Global Burden of Disease Study 2010. The Lancet Global Health. 2013; 1(5): e259–281. doi: 10.1016/S2214-109X(13)70089-5 25104492 PMC4181351

[3] Mutai H, Furukawa T, Araki K, Misawa K, Hanihara T. Factors associated with functional recovery and home discharge in stroke patients admitted to a convalescent rehabilitation ward. Geriatrics & Gerontology International. 2012; 12(2): 215–222. doi: 10.1111/j.1447-0594.2011.00747.x 21929733

[4] Van der Cruyssen K, Vereeck L, Saeys W, Remmen R. Prognostic factors for discharge destination after acute stroke: a comprehensive literature review. Disability and Rehabilitation. 2015; 37(14): 1214–1227. doi: 10.3109/09638288.2014.961655 25250810

[5] Thorpe ER, Garrett KB, Smith AM, Reneker JC, Phillips RS. Outcome Measure Scores Predict Discharge Destination in Patients With Acute and Subacute Stroke: A Systematic Review and Series of Meta-analyses. Journal of Neurologic Physical Therapy. 2018; 42(1): 2–11. doi: 10.1097/NPT.0000000000000211 29232307

[6] Chumney D, Nollinger K, Shesko K, Skop K, Spencer M, Newton RA. Ability of Functional Independence Measure to accurately predict functional outcome of stroke-specific population: systematic review. Journal of Rehabilitation Research and Development. 2010; 47(1): 17–29. doi: 10.1682/jrrd.2009.08.0140 20437324

[7] Data management service of the Uniform Data System for Medical R, the Center for Functional Assessment R. Guide for use of the uniform data set for medical rehabilitation. version 3.0 ed.: State University of New York at Buffalo; 1990.

[8] Keith RA, Granger CV, Hamilton BB, Sherwin FS. The functional independence measure: a new tool for rehabilitation. Advances in Clinical Rehabilitation. 1987; 1: 6–18. 3503663

[9] Inouye M. Predicting models of outcome stratified by age after first stroke rehabilitation in Japan. American Journal of Physical Medicine & Rehabilitation. 2001; 80(8): 586–591. doi: 10.1097/00002060-200108000-00008 11475479

[10] Fujiwara T, Liu M, Tsuji T, Sonoda S, Mizuno K, Akaboshi K, et al. Development of a new measure to assess trunk impairment after stroke (trunk impairment scale): its psychometric properties. American Journal of Physical Medicine & Rehabilitation. 2004; 83(9): 681–688. doi: 10.1097/01.phm.0000137308.10562.20 15314532

[11] Tsuji T, Liu M, Sonoda S, Domen K, Chino N. The stroke impairment assessment set: its internal consistency and predictive validity. Archives of Physical Medicine and Rehabilitation. 2000; 81(7): 863–868. doi: 10.1053/apmr.2000.6275 10895996

[12] Liu M, Domen K, Chino N. Comorbidity measures for stroke outcome research: a preliminary study. Archives of Physical Medicine and Rehabilitation. 1997; 78(2): 166–172. doi: 10.1016/s0003-9993(97)90259-8 9041898

[13] Moon S, Ahmadnezhad P, Song H-J, Thompson J, Kipp K, Akinwuntan AE, et al. Artificial neural networks in neurorehabilitation: A scoping review. NeuroRehabilitation. 2020; 46(3): 259–269. doi: 10.3233/NRE-192996 32250332

[14] Deo RC. Machine Learning in Medicine. Circulation. 2015; 132(20): 1920–1930. doi: 10.1161/CIRCULATIONAHA.115.001593 26572668 PMC5831252

[15] Li X, Pan X, Jiang C, Wu M, Liu Y, Wang F, et al. Predicting 6-Month Unfavorable Outcome of Acute Ischemic Stroke Using Machine Learning. Frontiers in Neurology. 2020; 11: 539509. doi: 10.3389/fneur.2020.539509 33329298 PMC7710984

[16] Lin W-Y, Chen C-H, Tseng Y-J, Tsai Y-T, Chang C-Y, Wang H-Y, et al. Predicting post-stroke activities of daily living through a machine learning-based approach on initiating rehabilitation. International Journal of Medical Informatics. 2018; 111: 159–164. doi: 10.1016/j.ijmedinf.2018.01.002 29425627

[17] Wang H-L, Hsu W-Y, Lee M-H, Weng H-H, Chang S-W, Yang J-T, et al. Automatic Machine-Learning-Based Outcome Prediction in Patients With Primary Intracerebral Hemorrhage. Frontiers in Neurology. 2019; 10: 910. doi: 10.3389/fneur.2019.00910 31496988 PMC6713018

[18] Heo J, Yoon JG, Park H, Kim YD, Nam HS, Heo JH. Machine Learning-Based Model for Prediction of Outcomes in Acute Stroke. Stroke. 2019; 50(5): 1263–1265. doi: 10.1161/STROKEAHA.118.024293 30890116

[19] Sonoda S, Chino N, Domen K, Saitoh E. Changes in impairment and disability from the third to the sixth month after stroke and its relationship evaluated by an artificial neural network. American Journal of Physical Medicine & Rehabilitation. 1997; 76(5): 395–400. doi: 10.1097/00002060-199709000-00010 9354494

[20] Sale P, Ferriero G, Ciabattoni L, Cortese AM, Ferracuti F, Romeo L, et al. Predicting Motor and Cognitive Improvement Through Machine Learning Algorithm in Human Subject that Underwent a Rehabilitation Treatment in the Early Stage of Stroke. Journal of Stroke and Cerebrovascular Diseases: The Official Journal of National Stroke Association. 2018; 27(11): 2962–2972. doi: 10.1016/j.jstrokecerebrovasdis.2018.06.021 30077601

[21] Shahid N, Rappon T, Berta W. Applications of artificial neural networks in health care organizational decision-making: A scoping review. PloS One. 2019; 14(2): e0212356. doi: 10.1371/journal.pone.0212356 30779785 PMC6380578

[22] Vapnik VN. An overview of statistical learning theory. IEEE transactions on neural networks. 1999; 10(5): 988–999. doi: 10.1109/72.788640 18252602

[23] Hasegawa K, Funatsu K. Non-linear modeling and chemical interpretation with aid of support vector machine and regression. Current Computer-Aided Drug Design. 2010; 6(1): 24–36. doi: 10.2174/157340910790980124 20370693

[24] Lancashire LJ, Lemetre C, Ball GR. An introduction to artificial neural networks in bioinformatics—application to complex microarray and mass spectrometry datasets in cancer studies. Briefings in Bioinformatics. 2009; 10(3): 315–329. doi: 10.1093/bib/bbp012 19307287

[25] Loh WY. Classification and regression trees. Wiley Interdisciplinary Reviews-Data Mining and Knowledge Discovery. 2011; 1(1): 14–23. doi: 10.1002/widm.8 PMC3329156 22523608

[26] Marshall RJ. The use of classification and regression trees in clinical epidemiology. J Clin Epidemiol. 2001; 54(6): 603–609. doi: 10.1016/s0895-4356(00)00344-9 11377121

[27] Henrard S, Speybroeck N, Hermans C. Classification and regression tree analysis vs. multivariable linear and logistic regression methods as statistical tools for studying haemophilia. Haemophilia. 2015; 21(6): 715–722. doi: 10.1111/hae.12778 26248714

[28] DeGregory KW, Kuiper P, DeSilvio T, Pleuss JD, Miller R, Roginski JW, et al. A review of machine learning in obesity. Obes Rev. 2018; 19(5): 668–685. doi: 10.1111/obr.12667 29426065 PMC8176949

[29] Rokach L. Ensemble-based classifiers. Artificial Intelligence Review. 2010; 33(1–2): 1–39. doi: 10.1007/s10462-009-9124-7

[30] Carino-Escobar RI, Valdés-Cristerna R, Carrillo-Mora P, Rodriguez-Barragan MA, Hernandez-Arenas C, Quinzaños-Fresnedo J, et al. Prognosis of stroke upper limb recovery with physiological variables using regression tree ensembles. J Neural Eng. 2021; 18(4). doi: 10.1088/1741-2552/abfc1e 33906163

[31] Wang W, Kiik M, Peek N, Curcin V, Marshall IJ, Rudd AG, et al. A systematic review of machine learning models for predicting outcomes of stroke with structured data. PLoS One. 2020; 15(6): e0234722. doi: 10.1371/journal.pone.0234722 . 32530947 PMC7292406

[32] Mainali S, Darsie ME, Smetana KS. Machine Learning in Action: Stroke Diagnosis and Outcome Prediction. Front Neurol. 2021; 12: 734345. doi: 10.3389/fneur.2021.734345 . 34938254 PMC8685212

[33] Lucas CG, Griffiths TL, Williams JJ, Kalish ML. A rational model of function learning. Psychonomic Bulletin & Review. 2015; 22(5): 1193–1215. doi: 10.3758/s13423-015-0808-5 25732094

[34] Lee SI, Mortazavi B, Hoffman HA, Lu DS, Li C, Paak BH, et al. A Prediction Model for Functional Outcomes in Spinal Cord Disorder Patients Using Gaussian Process Regression. IEEE journal of biomedical and health informatics. 2016; 20(1): 91–99. doi: 10.1109/JBHI.2014.2372777 25423659

[35] Stegle O, Fallert SV, MacKay DJC, Brage S. Gaussian process robust regression for noisy heart rate data. IEEE transactions on bio-medical engineering. 2008; 55(9): 2143–2151. doi: 10.1109/TBME.2008.923118 18713683

[36] Lee SI, Adans-Dester C, Obrien A, Vergara G, Black-Schaffer RM, Zafonte R, et al. Predicting and Monitoring Upper-Limb Rehabilitation Outcomes Using Clinical and Wearable Sensor Data in Brain Injury Survivors. IEEE transactions on bio-medical engineering. 2020; PP. 10.1109/TBME.2020.3027853. PMC8723794 32997621

[37] Hope TMH, Seghier ML, Leff AP, Price CJ. Predicting outcome and recovery after stroke with lesions extracted from MRI images. NeuroImage: Clinical. 2013; 2: 424–433. doi: 10.1016/j.nicl.2013.03.005 24179796 PMC3778268

[38] Rondina JM, Filippone M, Girolami M, Ward NS. Decoding post-stroke motor function from structural brain imaging. NeuroImage: Clinical. 2016; 12: 372–380. doi: 10.1016/j.nicl.2016.07.014 27595065 PMC4995603

[39] World Medical A. World Medical Association Declaration of Helsinki: ethical principles for medical research involving human subjects. JAMA. 2013; 310(20): 2191–2194. doi: 10.1001/jama.2013.281053 24141714

[40] Liu M, Sonoda S, Domen K. Stroke Impairment Assessment Set (SIAS) and Functional Independence Measure (FIM) and their practical use. In: Chino N, ed. Functional Assessment of Stroke Patients: Practical Aspects of SIAS and FIM. Tokyo: SplingerVerlag; 1997.

[41] Tsuji T, Sonoda S, Domen K, Saitoh E, Liu M, Chino N. ADL structure for stroke patients in Japan based on the functional independence measure. American Journal of Physical Medicine & Rehabilitation. 1995; 74(6): 432–438. doi: 10.1097/00002060-199511000-00007 8534387

[42] Yamada S, Liu M, Hase K, Tanaka N, Fujiwara T, Tsuji T, et al. Development of a short version of the motor FIM for use in long-term care settings. Journal of Rehabilitation Medicine. 2006; 38(1): 50–56. doi: 10.1080/16501970510044034 16548088

[43] Ellis C, Hyacinth HI, Beckett J, Feng W, Chimowitz M, Ovbiagele B, et al. Racial/Ethnic differences in poststroke rehabilitation outcomes. Stroke Research and Treatment. 2014; 2014: 950746. doi: 10.1155/2014/950746 25028619 PMC4084586

[44] Meyer MJ, Pereira S, McClure A, Teasell R, Thind A, Koval J, et al. A systematic review of studies reporting multivariable models to predict functional outcomes after post-stroke inpatient rehabilitation. Disability and Rehabilitation. 2015; 37(15): 1316–1323. doi: 10.3109/09638288.2014.963706 25250807

[45] Seeger M. Gaussian processes for machine learning. International Journal of Neural Systems. 2004; 14(2): 69–106. doi: 10.1142/S0129065704001899 15112367

[46] Oczkowski WJ, Barreca S. Neural network modeling accurately predicts the functional outcome of stroke survivors with moderate disabilities. Archives of Physical Medicine and Rehabilitation. 1997; 78(4): 340–345. doi: 10.1016/s0003-9993(97)90222-7 9111450

[47] Belliveau T, Jette AM, Seetharama S, Axt J, Rosenblum D, Larose D, et al. Developing Artificial Neural Network Models to Predict Functioning One Year After Traumatic Spinal Cord Injury. Archives of Physical Medicine and Rehabilitation. 2016; 97(10): 1663–1668.e1663. doi: 10.1016/j.apmr.2016.04.014 27208647

[48] Rodriguez JD, Perez A, Lozano JA. Sensitivity Analysis of k-Fold Cross Validation in Prediction Error Estimation. IEEE Transactions on Pattern Analysis and Machine Intelligence. 2010; 32(3): 569–575. doi: 10.1109/TPAMI.2009.187 20075479

[49] Waldmann P. On the Use of the Pearson Correlation Coefficient for Model Evaluation in Genome-Wide Prediction. Frontiers in Genetics. 2019; 10: 899. doi: 10.3389/fgene.2019.00899 31632436 PMC6781837

[50] Kotsiantis S. Supervised Machine Learning: A Review of Classification Techniques. Informatica (Slovenia). 2007; 31: 249–268.

[51] Raudys SJ, Jain AK. Small sample size effects in statistical pattern recognition: recommendations for practitioners. IEEE Transactions on Pattern Analysis and Machine Intelligence. 1991; 13(3): 252–264. doi: 10.1109/34.75512

[52] Sargent DJ. Comparison of artificial neural networks with other statistical approaches: results from medical data sets. Cancer. 2001; 91(8 Suppl): 1636–1642. doi: 10.1002/1097-0142(20010415)91:8+&lt;1636::aid-cncr1176&gt;3.0.co;2-d 11309761

[53] Hu X, Cammann H, Meyer H-A, Miller K, Jung K, Stephan C. Artificial neural networks and prostate cancer—tools for diagnosis and management. Nature Reviews Urology. 2013; 10(3): 174–182. doi: 10.1038/nrurol.2013.9 23399728

[54] Gómez-Moreno H, Gil-Jiménez P, Lafuente-Arroyo S, López-Sastre R, Maldonado-Bascón S. A "salt and pepper" noise reduction scheme for digital images based on Support Vector Machines classification and regression. TheScientificWorldJournal. 2014; 2014: 826405. doi: 10.1155/2014/826405 25202739 PMC4151368

[55] Forouzanfar MH, Foreman KJ, Delossantos AM, Lozano R, Lopez AD, Murray CJL, et al. Breast and cervical cancer in 187 countries between 1980 and 2010: a systematic analysis. Lancet (London, England). 2011; 378(9801): 1461–1484. doi: 10.1016/S0140-6736(11)61351-2 21924486

[56] Nii M, Maeda K, Wakabayashi H, Nishioka S, Tanaka A. Nutritional Improvement and Energy Intake Are Associated with Functional Recovery in Patients after Cerebrovascular Disorders. Journal of Stroke and Cerebrovascular Diseases: The Official Journal of National Stroke Association. 2016; 25(1): 57–62. doi: 10.1016/j.jstrokecerebrovasdis.2015.08.033 26409716

[57] Kwakkel G, Veerbeek JM, van Wegen EE, Nijland R, Harmeling-van der Wel BC, Dippel DW. Predictive value of the NIHSS for ADL outcome after ischemic hemispheric stroke: does timing of early assessment matter? J Neurol Sci. 2010; 294(1–2): 57–61. doi: 10.1016/j.jns.2010.04.004 20439108

[58] Nawabi J, Kniep H, Elsayed S, Friedrich C, Sporns P, Rusche T, et al. Imaging-Based Outcome Prediction of Acute Intracerebral Hemorrhage. Transl Stroke Res. 2021; 12(6): 958–967. doi: 10.1007/s12975-021-00891-8 33547592 PMC8557152

