# Supplementary material for: Improvement of predictive accuracies of functional outcomes after subacute stroke inpatient rehabilitation by machine learning models
Source: PLoS One. 2023 May 26;18(5):e0286269. doi: 10.1371/journal.pone.0286269 (PMC10218720; doi:10.1371/journal.pone.0286269)
Supplement: S1 Table — SE: standard error; Since Onset: days since onset, FIM: Functional Independence Measure. The aim of our study was to compare the predictive accuracies of a conventional stepwise linear regression (SLR) model and five machine learning models, Regression Tree, Ensemble Learning, Artificial Neural Network, Support Vector Regression, and Gaussian Process Regression. This study built the prognostic models for Activities of Daily Living (ADL) with the Functional Independence Measure (FIM), one of the methods for evaluating ADL. Discharge FIM motor scores, FIM cognitive scores, and FIM total scores were predicted. FIM gain is calculated by subtracting the scores at admission from those at the time of discharge. FIM motor gain, FIM cognitive gain, and FIM total gain were also predicted. A total of 1,046 subacute stroke patients who underwent inpatient rehabilitation participated in the present study. Patient information including age, sex, days since onset, admission and discharge FIM scores, a history of stroke, and transfer to other hospitals was gathered. Statistical analysis was performed with MATLAB software, version 2022a (The Mathworks, Natick, MA, USA). These predictive models were built with these participants’ information and 10-fold cross-validation. S1 Table shows the factors selected by the SLR model, and each value shows the intercept and coefficients. (DOCX) [file pone.0286269.s001.docx]

|  | Intercept | | | | Age | | | | Since Onset | | | | FIM cognitive scores on admission | | | | FIM total scores on admission | | | |
| --- | --- | --- | --- | --- | --- | --- | --- | --- | --- | --- | --- | --- | --- | --- | --- | --- | --- | --- | --- | --- |
| Predictive variable | Estimate | SE | t | *p* - value | Estimate | SE | t | *p* - value | Estimate | SE | t | *p* - value | Estimate | SE | t | *p* - value | Estimate | SE | t | *p* - value |
| FIM Motor scores  at discharge | 62.25 | 3.36 | 18.52 | *p* < 0.001 | -0.30 | 0.03 | -9.14 | *p* < 0.001 | -0.24 | 0.04 | -6.49 | *p* < 0.001 |  |  |  |  | 0.55 | 0.02 | 31.72 | *p* < 0.001 |
| FIM Cognitive scores  at discharge | 19.58 | 1.09 | 18.00 | *p* < 0.001 | -0.09 | 0.01 | -8.25 | *p* < 0.001 | -0.06 | 0.01 | -5.09 | *p* < 0.001 | 0.69 | 0.02 | 37.86 | *p* < 0.001 |  |  |  |  |
| FIM Total scores  at discharge | 80.97 | 4.22 | 19.19 | *p* < 0.001 | -0.36 | 0.04 | -8.95 | *p* < 0.001 | -0.32 | 0.04 | -7.27 | *p* < 0.001 | 0.69 | 0.11 | 6.11 | *p* < 0.001 | 0.55 | 0.04 | 15.51 | *p* < 0.001 |
| FIM Motor  gain | 60.08 | 3.46 | 17.38 | *p* < 0.001 | -0.29 | 0.03 | -8.83 | *p* < 0.001 | -0.22 | 0.04 | -5.96 | *p* < 0.001 | 1.16 | 0.10 | 12.04 | *p* < 0.001 | -0.48 | 0.03 | -16.19 | *p* < 0.001 |
| FIM Cognitive gain | 19.73 | 1.09 | 18.03 | *p* < 0.001 | -0.09 | 0.01 | -8.73 | *p* < 0.001 | -0.06 | 0.01 | -5.10 | *p* < 0.001 | -0.36 | 0.03 | -12.08 | *p* < 0.001 | 0.02 | 0.01 | 1.86 | 0.064 |
| FIM Total  gain | 81.03 | 4.12 | 19.66 | *p* < 0.001 | -0.38 | 0.04 | -9.67 | *p* < 0.001 | -0.31 | 0.05 | -6.73 | *p* < 0.001 | 0.75 | 0.12 | 6.55 | *p* < 0.001 | -0.46 | 0.04 | -12.32 | *p* < 0.001 |
